# Nutritional and Psychosocial Intervention to Improve the Self-Concept of Body Image and Increase the Self-Esteem of Overweight and Obese Individuals: A Quasi-Experimental Study

**DOI:** 10.3390/nu16162708

**Published:** 2024-08-14

**Authors:** Elvira González-Fernández, Raquel Xandri-Martínez, Magdalena Gómez-Díaz, Julia Navas-López

**Affiliations:** 1 Faculty of Pharmacy and Nutrition, Catholic University of Murcia (UCAM), Campus de Los Jerónimos s/n, 30107 Murcia, Spain; egonzalez495@alu.ucam.edu (E.G.-F.); jnavas@ucam.edu (J.N.-L.); 2 Faculty of Education, Catholic University of Murcia (UCAM), Campus de Los Jerónimos s/n, 30107 Murcia, Spain; rxandri@ucam.edu; 3 Faculty of Nursing, Catholic University of Murcia (UCAM), Campus de Los Jerónimos s/n, 30107 Murcia, Spain

**Keywords:** nutrition therapy, psychosocial intervention, overweight, obesity, body image, self-esteem, interdisciplinary study

## Abstract

Negative habits persist in contemporary society that can sometimes result in overweight or the deterioration of body image. This study aimed to assess the suitability of a nutritional and psychosocial intervention as part of an interdisciplinary approach to improve the perception of body image and increase the self-esteem of individuals who are overweight or obese. A total of 55 participants (25 men and 30 women) were included in this quasi-experimental intervention study. Measurements were taken as part of an ambulatory treatment to obtain values for weight, self-esteem, and body image perception using the Rosenberg scale and the Body Self-Esteem scale. At the end of the intervention and after one year, the weight reductions reached an average of 13.4 kg, positive self-image perception improved from a mean of 88.73 at pretest to 148.02 at follow-up, and self-esteem improved from a mean of 22.6 to 32.6. These were all statistically significant changes (*p* < 0.001). The model is effective in terms of weight reduction, together with improved levels of self-esteem and favorable perceptions of body image.

## 1. Introduction

Many different international organizations, from the area of global health (World Health Organization, WHO) to social and economic areas (Organization for Economic Co-operation and Development, OECD), have warned about the serious global problems relating to nutritional disorders concerning overweight and obesity [1,2,3].

Around 3.5 million people die each year due to health problems associated with obesity and/or overweight, a statistical figure that is expected to double by 2030 (around 7 million casualties). Furthermore, also by 2030, it is estimated that 23.3 million deaths will be attributable to cardiovascular disorders, most of which will be avoidable, due to sedentarism and obesity [4].

Likewise, the current data available tend to point to the growing trend of other, although equally serious, health problems associated with the presence of overweight and/or obesity [5,6,7,8], just as other data continue to highlight the seriousness of the increasing levels of cardiovascular disorders associated with these overweight or obesity problems [9,10,11].

In addition, we cannot forget the psychological problems that may be associated with overweight and/or obesity, from anxiety and/or depressive disorders or eating disorders to suicidal ideation or behaviors, sometimes as a consequence of low levels of self-esteem and/or distorted perceptions of body image [12,13,14,15,16].

Therefore, implementing preventive measures and/or early interventions, as well as building holistic therapeutic models aimed at eradicating inadequate habits to thereby deter behaviors that promote overweight and/or obesity, could be useful in terms of guaranteeing basic health standards for the population of any country, given the global nature of the problem described.

According to the above reasoning, there seems to be a sufficient consensus to consider that the variables involved in eating habits (family, educational, and social) are closely related to the emotions and moods of individuals and to current social influences [17,18,19,20]. Thus, disciplines such as psychology have great potential in terms of contributing theoretical and practical knowledge to provide different therapeutic proposals and offer adequate responses to deal with these nutritional and diet-related disorders. For example, adequate nutritional education or psychoeducation regarding emotional management and the use of food could be established for this purpose.

From this perspective, concepts such as emotional nutrition (defined as a type of eating behavior in which nutrients are chosen as a function of a specific mood) or underlying emotional conflicts coincident with maintaining specific eating habits are important core aspects that that need careful evaluation and intervention. For this, psychological approaches have been raised as fundamental tools in multidisciplinary approaches for the treatment of overweight and obesity [21].

Thus, given the preceding arguments, a new scientific discipline has been created that, through the combination of knowledge from the areas of psychology and nutrition, offers comprehensive models for a pluralistic approach. These models are known as psycho-nutritional models. They require two different professional interventions. The first of these consists of a nutritional approach (with a corresponding health professional who has accredited training in this area), followed by a psychological approach administered by corresponding professionals with psychosocial expertise. The most notable virtue of these models is that they are able to provide a specific answer to the problems for which an individual user seeks therapeutic intervention, while also addressing the underlying causes that have led to their current health status. In this way, a comprehensive and holistic approach is established that allows for the best contextualization of the specific situation, specifically considering the person experiencing it [22]. 

The psycho-nutritional model is based on a physical and emotional approach that seeks to evaluate and contextualize the specific importance of every self-acceptance and self-perception variable for each individual case relevant to each user.

This study aimed to examine the effect of a nutritional and psychosocial intervention on self-esteem and self-perceived body image, comparing the data obtained in the pre-intervention assessment with the results obtained directly after the intervention and one year after the intervention.

## 2. Materials and Methods

### 2.1. Design

This was a quasi-experimental study that included repeated pretest–posttest measurements of a single group, lacked a group control, and was carried out across three differentiated time periods (pretest, posttest, and follow-up, which was a year after the end of the therapeutic intervention).

The objectives were specified before the data were collected, the analysis plan was pre-specified, and any data-driven analyses were clearly identified and discussed appropriately.

### 2.2. Participants

The 55 final participants (25 men and 30 women) were aged between 25 and 70 years old, with a mean age of 45.98 years (with a standard deviation of 10.9 years). 

The dependent variables were weight, self-esteem, and self-perception of body image, while the independent variables were the moment in time in which the intervention took place.

### 2.3. Instruments

A digital floor scale was used to record the weight of the participants, enabling an objective assessment of their weight. Likewise, to measure the rest of the dependent variables, the Rosenberg self-esteem scale (EAR scale, 2007 Spanish adaptation) [23] and the Body Self-Esteem (EAC) scale by Peris and Maganto [24] were utilized. Both instruments have good levels of reliability (between 0.82 and 0.85 for the EAR scale and between 0.92 and 0.96 for the EAC scale). For both, a higher score indicates higher self-esteem. Likewise, a portable computer was utilized to store all of the measurement data collected throughout the study, and the data were processed with SPSS v.25.

### 2.4. Procedure

The procedure was implemented in such a way that significant initial achievements concerning overweight and obesity were obtained through the use of the designed psycho-nutritional program. This intervention began in 2018, and the data were collected until 2020. These data were obtained by asking for the voluntary, anonymous, and confidential participation of users who visited a private clinical nutrition center in Galicia (Spain) to ask for services relating to the treatment of overweight and obesity. The intervention was performed by a psychologist who was also a nutritionist. 

Thus, the users were informed that they would be assigned a numerical tag associated with their data, which were collected across three separate points in time (before the intervention—pretest; at the end of the intervention—posttest; and a year after discharge—follow-up). Before information collection, the participants were informed in detail about the objectives and the content of the study, after which they signed the informed consent form. At all times, the confidentiality of the collected information was ensured according to the ethical guidelines of the Declaration of Helsinki and with approval from the Ethics Committee of the Catholic University of Murcia, Spain (CE072205). 

The number of kilograms to be lost in each case was specifically agreed upon with the users, who were also informed that the intervention would be established with between 15 and 20 therapeutic sessions. These were weekly sessions through which weight reductions between 0.5 and 1 kg could be obtained.

For this, a holistic approach was used, as already mentioned, the intention of which was to dynamize the psycho-nutritional intervention and transfer (with the therapist’s empathy) the appropriate level of adherence to the model. Thus, the therapeutic work was developed from the perspective of serving a functional role beyond that which could be developed by a passive broadcaster of instructions. All of these aspects favored the transmission of permanent support throughout the entire process and promoted adequate motivation by providing nutritional pedagogy concerning food management, through which we were able to create specific diets for each user. 

Thus, the consolidation of new healthy eating habits, which consisted of reducing saturated fat consumption, balancing carbohydrates, and correctly distributing proteins, together with regularly practicing physical activity, could result in the desired achievements.

Lastly, measurements were taken relative to the three therapeutic periods mentioned, and they were collected in a spreadsheet for posterior statistical treatment, both descriptive and inferential. The systematization of the model used can be observed in the table below (Table 1). 

## 3. Results

A descriptive statistical analysis was performed, and the different fundamental characteristics of the variables were observed (those relative to the central tendencies, dispersion, bias, and type of distribution). Next, the normality of the distributions was analyzed for each of the variables of interest for the subsequent application of the inferential approach, with the most convenient parametric (or non-parametric) test utilized.

Table 2 shows the descriptive statistics analyzed related to the dependent variables of interest (weight, body self-esteem, and self-perception).

Observing the results allows us to point out the average weight reductions and the average increases in the self-esteem and body self-perception variables at a descriptive level.

Table 3 below shows the Kolmogorov–Smirnov test results.

As the data did not have a normal distribution, non-parametric tests were applied. Thus, given that three experimental conditions were available for each of the cases (pretest, posttest, follow-up), Friedman’s chi-square test for related samples was used. The results of this test are shown in Table 4 for all variables of interest. 

Given the trends described in the descriptive analysis, in which weight reductions were observed throughout the therapeutic process, as derived from the corresponding inferences, the results were statistically significant, as shown by the significance values obtained. The same was true for the statistically significant increases in both self-esteem and self-perceived body image.

Table 5 below shows that, between the different therapeutic periods, there were significant differences after the corresponding inferential analysis.

We can see that in all pairwise comparisons analyzed (except in the case of the comparison of posttest weight–annual follow-up weight), statistically significant differences occurred. 

Thus, the staging derived from the therapeutic intervention continued to show statistical significance, even one year after the intervention, since, in each of the cases, if we compared the pretest measurement with the follow-up measurement, we continued to find benefits (significant weight reductions, increased self-esteem, and improved self-perception of body image).

## 4. Discussion and Conclusions

After the end of this study, which intended to verify the efficacy of a therapeutic model based on a nutritional and psychosocial intervention, different conclusions could be made. The weight reductions at the end of the intervention, as well as at follow-up (a year after the end of the intervention), were significant and maintained over time, with a mean quantified reduction noted in both measurements. However, when comparing the posttest with the follow-up, there were no significant differences. We propose that new nutritional habits and healthier lifestyle habits were consolidated.

These results agree with those of other studies that have used nutritional and psychosocial approaches, in which beneficial effects were obtained with respect to a reduction in weight, which was also sustained over time [22,25,26]. 

As for the self-esteem variable, the trend was also positive, as the results indicated that, after the intervention, increased levels were found at both the end of the therapeutic approach and during the follow-up phase, obtaining highly significant values. Nevertheless, it is also true that although great increases in the variable mentioned were obtained, both after the intervention and during follow-up, these results did not seem to be stable over time for the weight variable. This finding could indicate that aspects such as self-esteem could be influenced by other intervening variables beyond the considerable reduction in weight. Thus, as time passes, the participants’ satisfaction with their individual achievements starts to lose its power in terms of positive reinforcement (although slightly). In this context, we refer to studies that have found a positive association between weight reductions and increased self-esteem [27,28,29]; however, these other studies did not find significant differences in self-esteem when comparing individuals with obesity with normoweight individuals [30].

Lastly, regarding the self-perception of body image, highly significant increases were observed, both in the posttest and in the follow-up phase. Likewise, we found studies that confirmed the association between obesity and a low self-perception of body image, as well as improvements in body image perceptions associated with weight reductions [31,32]. Nevertheless, there are different studies that have described a differential set of nuances: some found that weight reductions did not improve the self-perception of body image; instead, negative associations shifted to specific areas that participants were not satisfied with [33]. Other works found that the relationship was persistent in women but not in men [34], while others did not find differences between women with obesity and normoweight women, pointing out that differences were found in the degree of dealing with this image, being passive in women with obesity, while those who were normoweight presented behaviors directed towards seeking adequate physical care [35]. 

As a whole, we can define the indicators derived from the results described as guarantors of the efficacy of the model utilized, which was designed to obtain significant reductions in weight, along with changes in habits and the maintenance of the objectives reached over time (in our case, a year after the end of the intervention). These results agree with those found by the different authors mentioned [26]. We believe that this type of intervention is important in preventing social influences on perceptions of body image based on comparisons with models that are not always healthy despite being qualified as ideal.

Nevertheless, certain limitations of the study must be pointed out. These include limitations related to the lack of heterogeneity of the study participants, from which we obtained the responses analyzed. For example, the different members of the sample lived in a very specific geographical area; furthermore, they were not selected through probabilistic sampling, and a control group was not used with which to compare the results obtained. Thus, from a prospective perspective, a more in-depth study is needed with greater control over the different variables and a longer reach in terms of time (more follow-up phases), as well as greater heterogeneity in the observations, to confirm or refute the conclusions derived from the results obtained in this research study.

## Figures and Tables

**Table 1:** Systematization of the nutrition and psychosocial intervention scheduled by session with corresponding objectives and tasks.

Type of Session	Number of Sessions	Duration	Objective	Tasks
Motivational and Adherence	2 sessions	1 h/day (2 h)	- Realistic solution relative to individual reality. - Specify small achievements (1 kg per week). - Compliance with menu guidelines.	- Active listening. - Weigh the patient. - Autogenic relaxation. - Reinforcement of imagination.
Homeostatic Balance and Initial Intervention	5 sessions	1 h/day (5 h)	- First contact with new therapeutic reality applied to the day-to-day life of the user. - Changes in attitudes. - Compliance with menu guidelines.	- Active listening. - Weigh the patient. - Focus health standards.
Applied Intervention	10 sessions	1 h/day (10 h)	- Previous consolidation of objectives. - Acquisition of healthy nutritional habits. - Acquisition of physical activity habit. - Compliance with menu guidelines.	- Strengthen resilience through previous achievements. - Assertiveness. - Weigh the patient.
Consolidation	2 sessions	1 h/day (2 h)	- Prevention of relapse.	- Reinforcement of imagination. - Weigh the patient.
End of Therapy	1 session	1 h/day (1 h)	- Cessation of therapy.	- Peace of mind during the closing of the session. - Specific period of time for expression of emotions. - Weigh the patient.

**Table 2:** Descriptive values for the variables weight, BMI, self-esteem, and body self-perception of participants as a function of the pretest, posttest, and follow-up after a year.

Variable	Characteristic	Statistical Value	Pretest	Posttest	Follow-Up
WEIGHT	Central tendency	Arithmetic mean Median	87.6 85	74.2 74	74.2 73
	Variability	Standard deviation	17.4	11.1	11.54
	Asymmetry	Asymmetric index	1.70	0.72	0.98
	Direction	Kurtosis coefficient	3.85	1.14	1.97
BMI	Central tendency	Arithmetic mean	30.77	26.15	26.14
	Variability	Standard deviation	30.08	26.23	26.03
	Asymmetry	Asymmetric index	4.56	3.14	3.25
	Direction	Kurtosis coefficient	0.82	0.03	0.11
SELF-ESTEEM	Central tendency	Arithmetic mean Median	22.6 23	35.3 35	32.6 32
	Variability	Standard deviation	5.44	3.09	3.44
	Asymmetry	Asymmetric index	−0.08	−0.13	0.94
	Direction	Kurtosis coefficient	−1.09	−0.88	−0.06
BODY SELF-PER.	Central tendency	Arithmetic mean Median	88.73 87	150.91 147	148.02 145
	Variability	Standard deviation	12.82	16.74	18.32
	Asymmetry	Asymmetric index	0.21	0.41	0.64
	Direction	Kurtosis coefficient	0.39	1.1	1.61

**Table 3:** Kolmogorov–Smirnov goodness-of-fit to test the assumption of the normality of the variables of weight, self-esteem, and body self-perception according to pretest, posttest, and follow-up after one year.

Variable	Pretest Value (*p*-Value)	Posttest Value (*p*-Value)	Follow-Up	Normality Result
Weight	0.13 (0.02)	0.11 (0.17)	0.13 (0.02)	Absence
Self-esteem	0.12 (0.06)	0.11 (0.08)	0.24 (0.00)	Absence
Body self-perception	0.1 (0.2)	0.11 (0.08)	0.13 (0.02)	Absence

Note: Confidence level at 95%, α = 0.05.

**Table 4:** Friedman’s test for related samples as a function of the three measurement times: pretest, posttest, and follow-up.

Variable	Friedman’s Test	*df*	*p*-Value of the Pre–Post–Follow-Up Comparison	Decision
(A) Weight	77.27	2	0.000	Existence of significant differences
(B) Self-esteem	109.028	2	0.000	Existence of significant differences
(C) Body self-perception	91.931	2	0.000	Existence of significant differences

Note: Confidence level at 95%, α = 0.05.

**Table 5:** Multiple comparisons derived from Friedman’s test according to the three measurement periods: pretest, posttest, and follow-up.

Variable	Comparison	Mid-Range (+/−)	*p*	Decision
Weight	Pre–Post	6.5/27.9	0.000	Existence of significant differences
	Pre–Follow-Up	3/28.94	0.000	Existence of significant differences
	Post–Follow-Up	22.1/20.9	0.908	Absence of significance
Self-esteem	Pre–Post	28/0	0.000	Existence of significant differences
	Pre–Follow-Up	27/0	0.000	Existence of significant differences
	Post–Follow-Up	2.5/20.96	0.000	Existence of significant differences
Body self-perception	Pre–Post	28/0	0.000	Existence of significant differences
	Pre–Follow-Up	28/0	0.000	Existence of significant differences
	Post–Follow-Up	11.8/22.6	0.000	Existence of significant differences

Note: Confidence level at 95%, α = 0.05.

## Data Availability

The datasets used and analyzed during the current study are available from the corresponding author upon reasonable request. The data are not publicly available due to the inclusion of information that could compromise the privacy of the research participants.
